# A Non-Contact Electronic Nose System Based on Off-Gas Response for Real-Time NH_4_^+^ Monitoring in Fermentation

**DOI:** 10.3390/s26123667

**Published:** 2026-06-08

**Authors:** Xiaoqin Zhang, Daqi Gao, Yuan Wang

**Affiliations:** 1 Department of Computer Science and Engineering, East China University of Science and Technology, Shanghai 200237, China; gaodaqi@ecust.edu.cn; 2 School of Bioengineering, East China University of Science and Technology, Shanghai 200237, China; y30230511@mail.ecust.edu.cn

**Keywords:** electronic nose, NH_4_^+^, non-contact monitoring, real-time online monitoring, fermentation off-gas, gentamicin fermentation

## Abstract

Real-time online monitoring of key parameters such as the ammonium nitrogen (NH_4_^+^) concentration during biological fermentation is important for the optimization of a biological fermentation process. Traditional offline detection technologies like spectrophotometry need contact sampling, with drawbacks of monitoring lag, risk of contamination, etc. In this work, taking the gentamicin fermentation process as an example, we developed an intelligent electronic nose non-contact monitoring system on the basis of fermentation off-gas signals. We captured the typical signals of off-gas and established a quantitative relationship between the signals and the NH_4_^+^ concentration in fermentation broth; the system then realized non-contact real-time monitoring. The whole system consists of a gas-switching module, a sensor array module, a signal-processing module, and an intelligent prediction module. The system adopts a five-phase gas switching strategy to suppress sensor drift in metal–oxide–semiconductor (MOS) sensors and a light neural network for prediction, which improve both prediction speed and accuracy. Experiments were conducted using a 5 L fermenter, and the prediction result was consistent with the offline measured value (coefficient of determination, R^2^ = 0.9871, root mean square error, RMSE = 0.0317 g/L). This technique provides a new method for the non-contact measurement of key fermentation parameters, and it can be expanded to other fermentations.

## 1. Introduction

In the fermentation process of gentamicin, NH_4_^+^ serves as the main nitrogen source, and its concentration directly regulates microbial metabolism and product synthesis [1]. Therefore, achieving online monitoring of its concentration is crucial for optimizing feeding control and enhancing production efficiency. The biosynthesis of gentamicin relies on tight coordination between nitrogen metabolism and carbon (energy) metabolism. Recent studies further reveal that microbial uptake and utilization of NH_4_^+^ are closely related to core pathways such as cellular energy metabolism [1]. Consequently, changes in NH_4_^+^ concentration in the fermentation broth directly affect the overall metabolic state of the cells. Based on this mechanism, changes in cellular metabolic state driven by fluctuations in NH_4_^+^ levels may be specifically reflected in the composition of volatile organic compounds (VOCs) in fermentation off-gas.

Currently, mainstream means for detecting NH_4_^+^ concentration in the fermentation industry, like spectrophotometry, are typical contact analytical techniques offline. They need physical sampling of the fermented broth, which is laborious and results in delays in monitoring; however, above all, frequent sampling will aggravate contamination risk in the fermentation tank. These intrinsic disadvantages make it very difficult for traditional methods to meet the requirements of real-time, non-contact, and online monitoring in modern fermentation technologies.

Electronic nose technology simulates the human sense of smell with an array of gas sensors with broad-spectrum cross-sensitivity to complex volatile components and responds to the complex volatile gas components to generate a unique “odor fingerprint”, which is an effective technique for qualitative and quantitative analysis of gases. On this basis, application of the electronic nose in the monitoring of fermentation processes also has its advantages: by detecting the response signal of fermentation off-gas, a quantitative correlation model between off-gas signal and the concentration of NH_4_^+^ in fermentation broth can be established, therefore realizing true non-contact indirect monitoring, completely avoiding contamination problems, and providing a new technical pathway for online long-term stable monitoring [2].

However, there are still many difficulties encountered when applying electronic noses to a complex industrial fermentation processing environment. For instance, the composition of fermentation off-gas is quite complex, and fluctuating temperature and humidity around the fermentation reactor create challenges for sensor stability. Popular MOS sensors are prone to baseline drift, which leads to gradual baseline shifts during long-term continuous operation, signal distortion, and a decrease in the monitoring precision of sensors [3]. The second is that the key difficulty preventing the application of this technology to industrial processes lies in extracting good and stable information from a dynamic response signal, and building effective models with the requirements of speed, precision, and the real-time prediction accuracy of data-driven models. In short, the long-term sensor signal stability is poor, and the real-time data prediction accuracy of the data-driven model is weak; these are the two greatest barriers.

To address these challenges, current research primarily unfolds along two complementary directions: the first is innovation in the sensing elements themselves [4], enhancing intrinsic performance through new materials (e.g., MOFs and their derivatives [5]) and novel structures (e.g., dual-active-site heterojunctions [6]); the second is system integration, which employs hardware–software co-design strategies to overcome drift and interference at the system level—an approach crucial for practical deployment in complex industrial settings. This study is grounded in the second direction, focusing on resolving the prominent challenges of long-term stable monitoring and real-time prediction for commercial electronic nose systems in real industrial fermentation scenarios through system-level hardware–software co-innovation.

Therefore, we designed an intelligent non-contact electronic nose system intended for industrial fermentation monitoring. It consists of four functional modules: a gas path control module, a sensor array module, a signal-processing module, and an intelligent prediction module. By pairing hardware strategies and software algorithms, they improve the reliability and accuracy of monitoring step by step. In this work, we propose an innovative five-phase gas switching strategy. We actively control the system at the hardware level to overcome the influence of sensor drift and extract robust information. Moreover, we employ a lightweight neural network prediction model to make high-precision, rapid predictions of off-gas fingerprint, which shows the concentration of the target on the monitor computer, guaranteeing the real-time response of the system.

The main contributions of this paper are as follows:

1. We proposed and verified a general non-contact monitoring solution; constructed a complete system throughout the off-gas sampling process, signal processing, and real-time prediction; and verified the technical practicability of the online monitoring of key metabolite concentrations in the tank using off-gas response.
2. We proposed a hardware strategy for the signal robustness for long-term stable operation through the initial calibration and a five-phase gas switching cycle; effectively eliminated the sensors’ baseline drift and environmental disturbance; and achieved long-term repeatability for the monitoring signals.
3. To achieve monitoring that combines high accuracy and real-time capability, we used an algorithm–system co-design. We deeply integrated a lightweight neural network prediction model with the five-phase gas switching system and thus achieved monitoring performance equal to that of offline standard methods as well as stable, real-time online responses in a 5 L gentamicin fermenter, thereby providing reliable technical support for intelligent monitoring of industrial fermentation.

## 2. Materials and Methods

### 2.1. Fermentation Experiments and Acquisition of Benchmark Data

To obtain off-gas response signals and concentration benchmark data of NH_4_^+^ in a fermentation broth for modeling and validation, our study conducted a gentamicin fermentation experiment. We selected Micromonospora echinospora 49-92S (preserved in our laboratory culture collection, East China University of Science and Technology, Shanghai, China) as the production strain, and the fermentation time was 144 h in a 5 L fermenter with a normal process. The fermentation environment was maintained at a fixed level by an automatic control device. Samples were collected every 12 h during the fermentation process, and the concentration of NH_4_^+^ in the fermenter broth was determined offline by spectrophotometry. The results served as the ground-truth data for later development and validation of the electronic nose model.

### 2.2. System Architecture and the Concept of Non-Contact Monitoring

**Figure 1:**
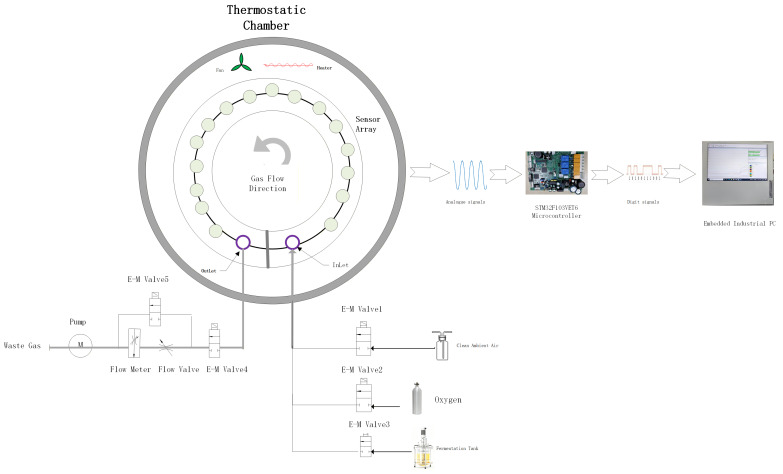
System architecture.

The system architecture is shown in Figure 1, which consists of a fermenter, off-gas collection unit, gas flow control unit, temperature-controlled sensing chamber, 16-channel MOS sensor array, data acquisition module, and host computer software. During the whole process, only the fermentation off-gas will be collected for measurement. Sensors are completely physically isolated from the fermentation broth, avoiding the risks from contact sampling, thereby meeting the requirements of long-term online measurement.

### 2.3. Five-Phase Gas Switching Strategy for Signal Robustness

**Figure 2:**
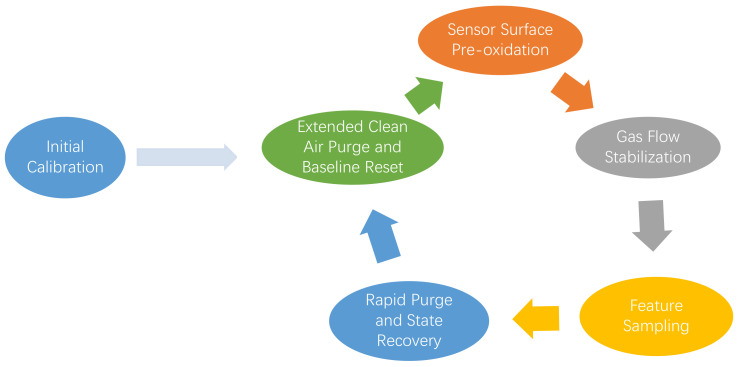
Initial calibration plus five-phase gas switching strategy.

It is well known that problems such as baseline drift, poor response repeatability, and high sensitivity of MOS sensors to environmental interference exist during long-term operation of sensors. To address these, we use an initial calibration plus a five-phase gas switching strategy of MOS sensors in this work (Figure 2).

#### 2.3.1. Initial Calibration

After the system is started up, clean air is first introduced for continuous preliminary calibrating for 9 h or 10 h. This enables the basic resistance of all sensors to stabilize in time fully and reach the set range, eliminates the initial state differences between sensors, provides a reference for long-term stable monitoring, etc.

#### 2.3.2. Five-Phase Gas Switching Process

Each monitoring cycle automatically triggers the following five steps in a fixed order.

1. Extended clean air purge and baseline resetting: clean air is purged for 185 s to purge the gas remanence from surfaces of the sensor components and uniformly reset the response baseline.
2. Sensor surface pre-oxidation: a 40-s purge is performed to prepare and stabilize the sensor surface (a detailed discussion on the gas source selection for this phase is provided in Section Rationale for Gas Source Selection in the Pre-Oxidation Phase).
3. Gas flow interval: keep gas flow stable for 10 s to remove gas flow disturbance caused by opening and closing the valve and enable stable sampling conditions.
4. Feature sampling: fermentation off-gas is introduced into the gas path for 55 s; when the response of the sensor drops to a steady-state value plateau, the steady-state value is recorded as the model feature.
5. Rapid purge and state recovery: switch to clean air for a fast 10 s purge, which rapidly desorbs gas from the sensor and returns it to baseline state and reads the current measurement cycle.

### 2.4. Hardware System Design

#### 2.4.1. Sensor Array

The core sensing unit is a 16-channel metal–oxide–semiconductor (MOS) sensor array. The selection of the sensor array took into careful consideration the characteristic volatile metabolites potentially present in the gentamicin fermentation off-gas (such as alcohols, ketones, organic acids, amines, sulfur-containing compounds, etc.). The selected commercial MOS sensors (e.g., TGS822, TGS826, TGS2602, TGS2620, etc.) exhibit high sensitivity and cross-sensitivity to multiple categories of these substances, ensuring the ability to capture the holistic VOC fingerprint that reflects the metabolic state. The array comprised commercial Figaro TGS-series MOS sensors (models: TGS813, TGS816, TGS821, TGS822, TGS823, TGS826, TGS830, TGS831, TGS832, TGS880, TGS2600, TGS2602, TGS2603, TGS2610, TGS2620) (Figaro Engineering Inc., Osaka, Japan).

**Figure 3:**
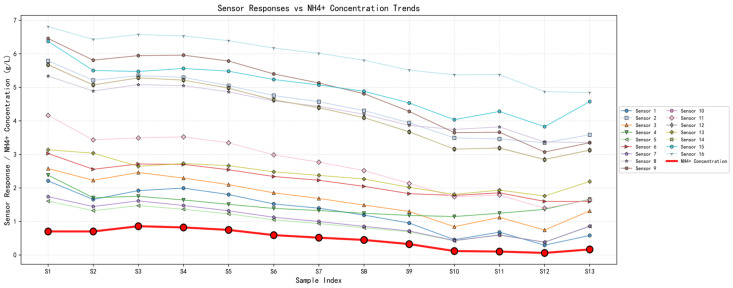
Steady-state peak responses of 16-channel sensors for 13 fermentation samples versus measured NH_4_^+^ concentration curves. Note: The responses of sensors #10 (TGS822), #12, and #14 (both TGS826) are highly similar, leading to overlapping traces. This results from intentional sensor redundancy and shared sensitivity to prevalent VOCs in the off-gas.

This configuration serves a dual purpose. First, it forms a broad-spectrum sensing platform. The selected sensors exhibit distinct yet partially overlapping sensitivity profiles towards a wide range of volatile organic compounds (VOCs), generating a collective, cross-reactive “fingerprint” of the fermentation off-gas. Second, the array incorporates intentional redundancy by including multiple units of the same model (e.g., TGS826). This is a standard reliability engineering measure for long-term monitoring, enhancing data robustness. Critically, the highly consistent response observed between these identical sensors (see Figure 3) provides empirical validation of the measurement system’s uniformity. It confirms that the temperature-controlled chamber (50 °C) and optimized gas flow dynamics create a homogeneous environment, ensuring that all sensors are exposed to virtually identical conditions.

#### 2.4.2. Temperature-Controlled Sensor Chamber

The chamber is made of stainless steel and contains a high-precision temperature control module for temperature control of sensors within a constant operating temperature of 50 °C, without being affected by temperature and humidity changes of sensors’ response.

#### 2.4.3. Gas Switching System

Consisting of a microcontroller, solenoid valves, and a mass flow controller, this system can automatically achieve switching between three different gas sources, clean air, pure oxygen, and fermentation off-gas, and it exactly control the flow rate and duration of each phase.

##### Gas Source and Conditioning

The “clean air” used in this system for purging and baseline resetting was sourced from the centralized laboratory air supply system, which provides compressed and filtered air. Its composition is consistent with clean ambient air. To ensure a stable measurement baseline, this gas is passed through the thermostatic sensor chamber (maintained at 50 °C) before reaching the sensor array, which stabilizes its temperature and effective humidity and reduces interference caused by moisture variations. This processed gas source is designed to provide a stable and repeatable reference for the sensor array. The resulting long-term baseline stability is demonstrated in Section Humidity Stability Strategy.

##### Flow Rate Specifications

The flow rates for each gas source were carefully controlled and kept constant throughout all experiments to ensure measurement consistency. During the five-phase switching cycle: (1) the clean air purge (Phases 1 and 5) was maintained at 6500 mL/min to ensure efficient removal of residual gases and rapid sensor recovery; (2) the oxygen supply (Phase 2) and fermentation off-gas introduction (Phase 4) were both set to 1000 mL/min. These flow rates were optimized to provide sufficient gas exchange for a reliable sensor response while maintaining a stable hydrodynamic environment in the sensing chamber. The use of a mass flow controller ensured precise and repeatable flow regulation across all monitoring cycles.

##### Rationale for Gas Source Selection in the Pre-Oxidation Phase

The gas path system is designed to allow for the flexible use of pure oxygen during the pre-oxidation phase (Phase 2). This functionality is reserved for potential future industrial applications, aimed at addressing complex and variable industrial environments. In such settings, pure oxygen can be utilized to rapidly bring the sensor response to a predetermined and stable baseline state.

However, in the controlled laboratory conditions of the present study, using the same dry, conditioned “clean air” (as described in Section Gas Source and Conditioning) for a 40-s purge during Phase 2 was sufficient to achieve a stable and reproducible baseline. This approach was deliberately chosen to ensure that the background oxygen concentration remained relatively constant from the extended clean air purge (Phase 1) through the pre-oxidation phase (Phase 2). Consequently, during the subsequent switch to the feature sampling phase (Phase 4, fermentation off-gas), the sensor response was not confounded by a drastic change in oxygen levels. This allows the observed signal to be reliably attributed to the VOCs in the fermentation off-gas rather than to interference from varying oxygen concentration.

##### Humidity Stability Strategy

To mitigate the known sensitivity of MOS sensors to humidity fluctuations, a system-level hardware strategy was implemented, centered on a temperature-controlled chamber maintained at 50 °C.

This approach stabilizes the effective humidity for two key reasons. First, maintaining a constant elevated temperature reduces water vapor adsorption on sensor surfaces, a principle supported by studies where heating measurement cavities to a similar temperature (e.g., 50 °C) effectively suppressed interference from adsorption–desorption effects [7,8]. Second, the “clean air” used for purging and baseline resetting is sourced from a dehumidified and filtered supply, providing a dry, stable reference gas that is further conditioned by the 50 °C chamber.

The combination of thermal conditioning of all gases and the use of a dry reference creates a stabilized measurement microenvironment. The long-term baseline stability achieved (CV < 3% for all 16 sensors throughout the experiment) serves as a key validation of the integrated effectiveness of the hardware strategies described in this section, including gas conditioning, flow control, and active humidity stabilization.

While the fermentation off-gas itself is not actively dehumidified prior to sampling, the combination of thermal conditioning and the use of a stable reference gas within a controlled hardware environment effectively mitigates the impact of humidity variations on the sensor array’s response. The high correlation between the stabilized off-gas “fingerprint” and NH_4_^+^ concentration (Section 3.2) confirms the robustness of the extracted features under this controlled measurement regime.

#### 2.4.4. Data Acquisition and Data Communication

The lower computer is developed by an STM32F103VET6 microcontroller (STMicroelectronics, Geneva, Switzerland) and is responsible for sensor signal acquisition, chamber temperature control, and gas path timing control. Raw data are subjected to a trimmed mean filter to remove noise and abnormal spikes in signals so as to stabilize the signals. The system uses a dual-serial-port independent data transmission structure: COM1 is used for the upper data transmission of the sensor and also realizes the related data control command transmission; COM2 is used for uploading environmental data to the upper computer and also transmits related control commands from the upper computer. So, the transmission of sensor data, environmental parameters, and control commands is completely independent, ensuring the real-time requirement and the reliable operation of the system.

### 2.5. Software Architecture and Real-Time Prediction Workflow

We developed supporting host computer software in this project. The major design idea of our software is to implement real-time online prediction for NH_4_^+^ concentration by integrating a lightweight neural network model and keeping the software versatile at the same time. To guarantee clarity and maintainability of the software architecture design, the software requirements analysis and design were modeled by using the RUP 4+1 model [9].

#### 2.5.1. System Functional Definition (Use Case Diagram)

**Figure 4:**
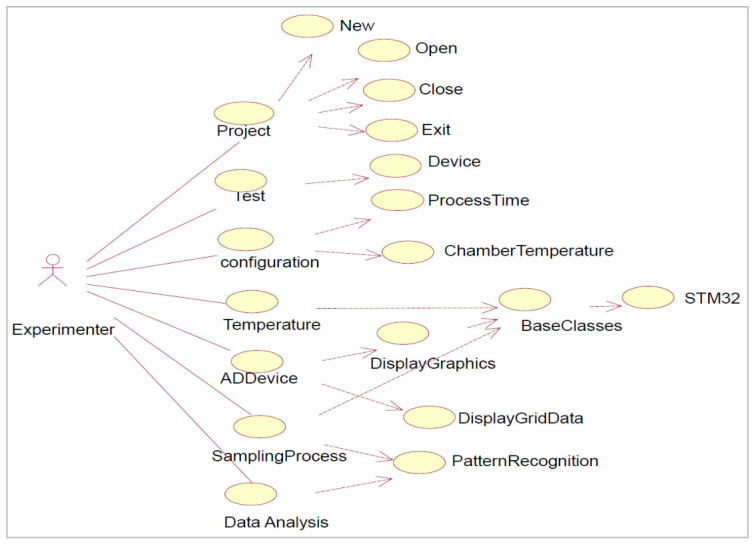
Use case diagram. Solid lines indicate the associations between actors and use cases. Dashed arrows with an open arrowhead denote the <<include>> dependencies between use cases.

Figure 4 shows how the functions of this software are designed around the basic operation of fermentation monitoring performed by laboratory technicians. The main functions of software include the following.

- The system parameter uses the sensor chamber temperature and control duration for each gas switching phase.
- The test gas switching equipment monitors whether the solenoid valves participating in the timing sequence of the gas switching are normal.
- The control five-phase gas switching switches the solenoid valves through the relay control, enable automatic switching among three gas source cases (clean air, oxygen, fermentation off-gas), and meet the working conditions of sensors at each case.
- To display data in real time, after starting sampling by operating system, the system automatically runs through the sampling process, to display the sensor curve, number value, and environmental parameter in real time.
- For NH_4_^+^ concentration prediction, we use feature extraction and saving, call the model to predict NH_4_^+^ concentration, and display the prediction result.

#### 2.5.2. System Static Design Structure

The system’s static design is organized on two levels: the components and the class diagram.

**Figure 5:**
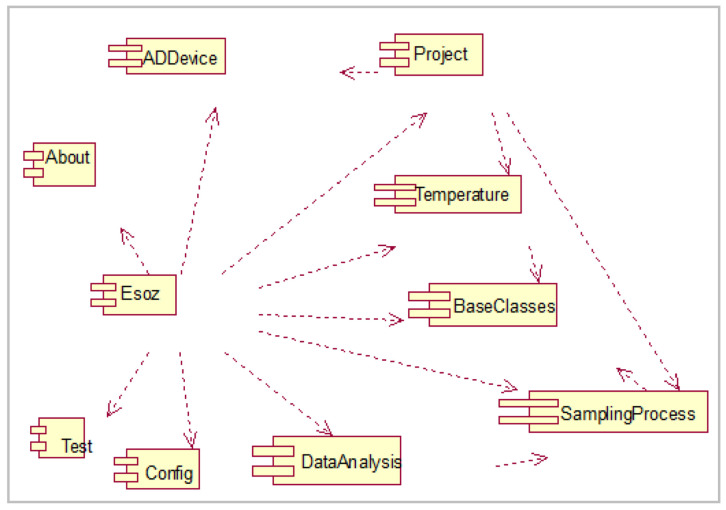
Component diagram. Dashed arrows with an open arrowhead denote dependencies between components.

**Figure 6:**
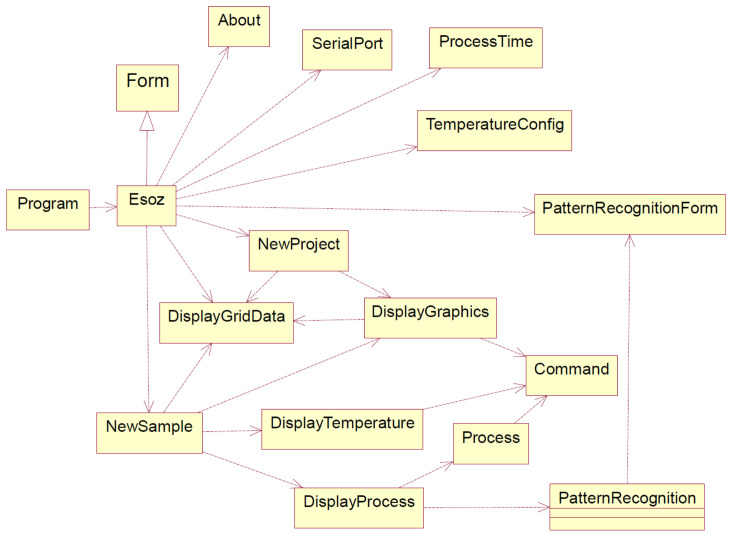
Class diagram.

- Component Diagram: As shown in Figure 5, the system software is clearly divided into six main modules with high cohesion and low coupling: the gas switching equipment test module, the five-phase cyclic gas switching control module, the sampling display module, the temperature humidity control module, the intelligent predictive module, the low-level service module, and so on. Modules communicate with each other through an interface and use multithreading technology for concurrent operations of every function module.
- Class Diagram: Figure 6 shows that each module function is realized by the cooperation of a set of specific classes. Specifically, the SerialPort class acts for the test module of gas switching equipment; the DisplayProcess class acts for the five-phase cyclic control of gas switching; the DisplayGraphics and DisplayGridData classes act for the sampling and displaying module; the DisplayTemperature class acts for the temperature and humidity monitoring module; the PatternRecognition class acts for the intelligent prediction module; and the Command class acts for the base service module. The class diagram reflects in detail the association and dependency of each main class, which serves as a blueprint for software implementation.

#### 2.5.3. System Deployment and Monitoring Interface

**Figure 7:**
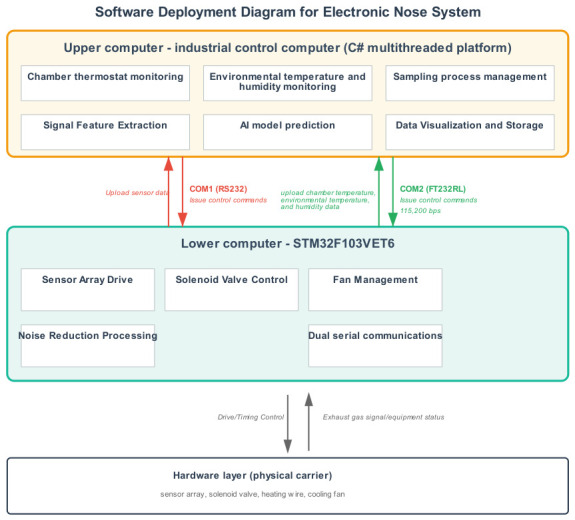
Deployment diagram.

Physical deployment of the system is shown in Figure 7, based on the lower computer/host computer collaborative model.

**Figure 8:**
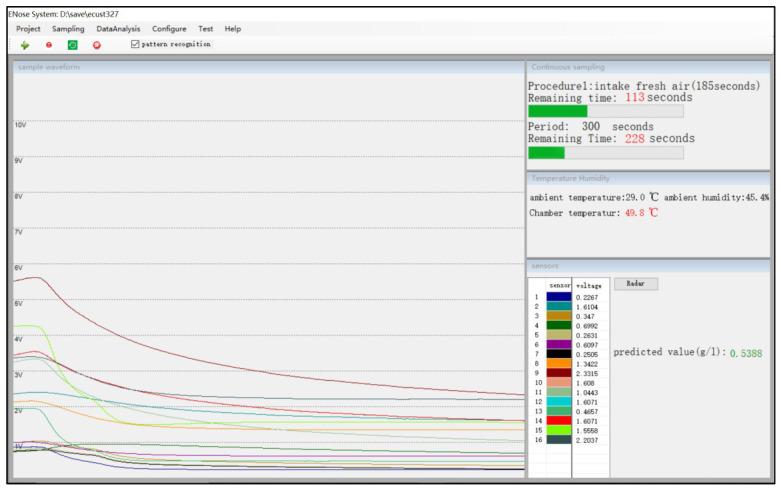
Host computer running interface.

- Lower Computer Deployment: Embedded firmware operates on an STM32F103VET6 microcontroller, directly managing sensor drivers, analog signal acquisition, temperature control, and real-time control of gas switching valves.
- Host Computer Deployment: The main control software, written in C#, runs on an industrial computer and uses C#’s ProcessStartInfo class to start a pre-trained simplified PyTorch neural network model, which was implemented in Python 3.7.1 with PyTorch 1.10.2. The monitoring interface (Figure 8) is clearly layered into five parts, detailed as follows.
  1. The gas switching phase area displays the current active gas path phase in real time.
  2. The sensor array response curve area plots the response curves of 16 sensors in real time.
  3. The real-time sensor response value area synchronously displays the real-time response values of 16 sensors.
  4. The environmental parameters display area shows the chamber temperature, room temperature, and room humidity in real time.
  5. The NH_4_^+^ concentration prediction area displays the NH_4_^+^ concentration-predicted current result.

#### 2.5.4. Real-Time Prediction Process

The software automatically completes the following steps in each sampling cycle to conduct automatic NH_4_^+^ concentration prediction.

1. Feature extraction: when the system is in Phase 5 (Rapid Purge and State Recovery), the DisplayProcess extracts steady-state peak values in 16 sensors to construct a feature vector.
2. NH_4_^+^ concentration prediction: when the system states are in Phase 1 (Extended clean air purge and baseline resetting), the PatternRecognition class invokes a lightweight PyTorch (version 1.10.2) NN model that has been pretrained to do the prediction, and the prediction results are updated instantly to the prediction area in the interface (Figure 8).

### 2.6. Feature Selection and Lightweight Neural Network Modeling

For the experimental data from the gentamicin fermentation process, in this section, we describe the criteria for picking out important features, the validation of data, and we give an elaborate introduction to the construction method and validation methods for a simple neural network model constructed for the real-time prediction of NH_4_^+^ concentration.

#### 2.6.1. Feature Selection Strategy and Correlation Validation

Various kinds of features, including static features, dynamic features, and geometric features, can be extracted from dynamic response curves of MOS sensors; the choice of features directly influences the accuracy and robustness of the model.

We took the peak response of the sensor in the steady-state plateau region of the dynamic response curve as the core features of modelling based on the following reasons:

- Compatibility with the Sensing Mechanism: the peak response is closely related to gas concentration, can realistically reflect changes of metabolism-related VOCs in off-gas, and can be reliably used [10,11].
- Stable signal: the peak response has a low coefficient of variation, is less affected by drift and fluctuations of the environment, and has better stability than that of dynamic and geometric features.

To verify the effectiveness of this feature, the Pearson correlation coefficient between the sensor’s steady-state peak response and NH_4_^+^ concentration was calculated to quantitatively analyze the correlation between the two [12,13,14].

#### 2.6.2. Framework for Building and Validating a Lightweight Neural Network Model

##### Model Construction Principles

Our study adopts a structurally simple single hidden-layer feed-forward NN. The structure is simple with few parameters, it helps against overfitting under small samples, and it has good nonlinear fitting properties (guaranteed by the Universal Approximation Theorem) [15,16,17,18]. A single hidden-layer feed-forward NN is able to capture the nonlinear mapping relationship between sensor features and NH_4_^+^ concentration, so the neural network meets the requirements of online inference.

##### Dataset Construction

This study collected a total of 13 valid data samples that cover the whole fermentation cycle. Each sample consists of the steady-state peak features of 16 sensors and true NH_4_^+^ values determined by spectrophotometry.

##### Lightweight Neural Network Architecture

The lightweight neural network has the following structure and key parameters.

- Input layer: 16 neurons (inputs are the peak values of the 16 sensors).
- Hidden layer: 12 neurons, activated with the ReLU activation function.
- Output layer: 1 neuron, the linear activation function, giving the predicted NH_4_^+^ concentration value (g/L) of the output layer.
- Optimizer: Adam, learning rate: 0.00002, epochs: 1000.
- Total number of parameters: 217, suitable for lightweight deployment.

##### Model Validation and Comparative Experiments

This section experimentally validates the advantages of the proposed lightweight neural network model. We compare it with Support Vector Regression (SVR) and Random Forest Regression (RF) using the same dataset, same input features, and a consistent evaluation protocol. The hyperparameters for SVR and RF were optimized via grid search, while the lightweight neural network’s architecture and hyperparameters were empirically preset. Model evaluation was conducted by using leave-one-out cross-validation [19,20]. The two evaluation indices, including the coefficient of determination (R^2^) and the root mean square error (RMSE), were used to evaluate the predictive accuracy and generalization capability of the model. Experimental comparison results and an analysis of model performance details are shown in Section 3.4.

## 3. Results

Long-term signal stability, off-gas response, NH_4_^+^ concentration predictive performance, and the performance of the model of the constructed non-contact electronic nose system in a 5 L fermentation system of gentamicin were examined in this section.

### 3.1. Sensor Baseline Stability Under the Five-Phase Gas Switching Strategy

For testing the long-term reliability of the system, the baseline stabilities of the 16-channel MOS sensor array were continuously tested for the five-phase gas switching strategy. In each monitoring cycle, the steady-state baseline values of the sensors were recorded in the third phase (gas flow stabilization phase), and their stability was evaluated by the coefficient of variation (CV) [21,22].

**Figure 9:**
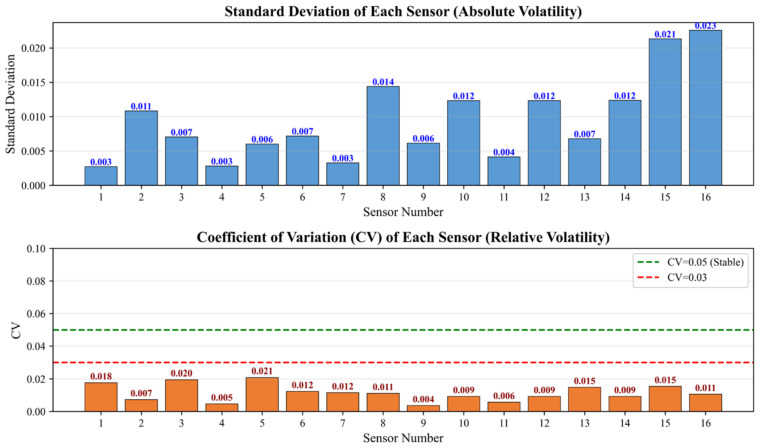
Coefficient of variation (CV) and standard deviation of sensor baselines under the five-phase gas switching strategy.

In Figure 9, the results of the 130 consecutive online monitoring cycles are presented. The CV of the baseline responses of all 16 sensors is below 3%, which is much lower than the predefined baseline stability limit of 5%.

These results prove that the initial calibration and five-phase gas switching strategy effectively suppresses sensor baseline drift and counteracts the impact of environmental variations on carrier gas conditions during prolonged operation. This ensures a stable, reproducible signal basis for accurate feature extraction and prediction.

### 3.2. Fermentation Off-Gas Response Features and Dataset

**Figure 10:**
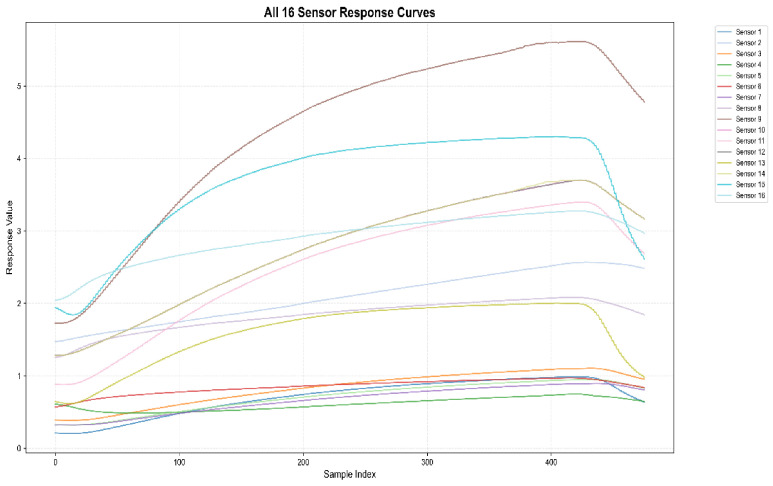
Dynamic response curves of a 16-channel MOS sensor array to gentamicin fermentation off-gas within one monitoring cycle (phase 4: the feature sampling phase).

In the feature sampling phase, fermentation off-gas was introduced. Upon introduction, the sensor responses rapidly increased and stabilized at a steady-state plateau. A significant variation in sensitivity and response amplitude was observed across the different sensors. This variation collectively forms a distinct off-gas “fingerprint” pattern, which can be used to discern the metabolic state (Figure 10).

According to the feature selection scheme mentioned in Section 2.6.1, the steady-state peak value of every sensor was selected as the input for modeling. Over the course of the fermentation, 13 effective samples were collected at 12-h intervals. Each sample comprised a 16-dimensional vector of sensor peak features and a corresponding NH_4_^+^ concentration value measured offline by spectrophotometry. The concentration ranges were from 0.1 to 0.8 g/L, which fully spanned the whole process of nitrogen source consumption, as shown in Figure 3.

**Figure 11:**
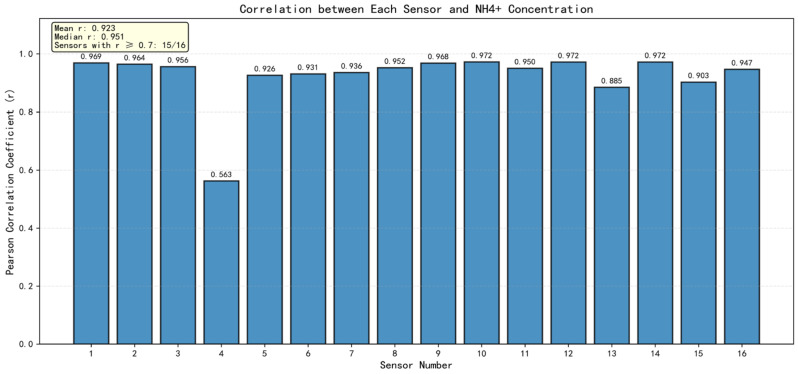
Pearson correlation coefficients between the steady-state peak features of 16 sensors and NH_4_^+^ concentration.

Figure 11 shows the results of Pearson correlation, which implies that 15 exhibited a correlation coefficient of ≥0.85 with the target concentration, with an average correlation coefficient of 0.923. The strong correlation of these features with the target concentration suggests that using the off-gas fingerprint to predict the concentration of NH_4_^+^ in the tank is feasible.

### 3.3. Predictive Performance of the Lightweight Neural Network for NH_4_^+^ Concentration

The lightweight single-hidden-layer feed-forward neural network (16-12-1 architecture described in Section 2.6.2) was evaluated using leave-one-out cross-validation (LOOCV) to rigorously assess its generalization performance given the small sample size (*n* = 13).

**Figure 12:**
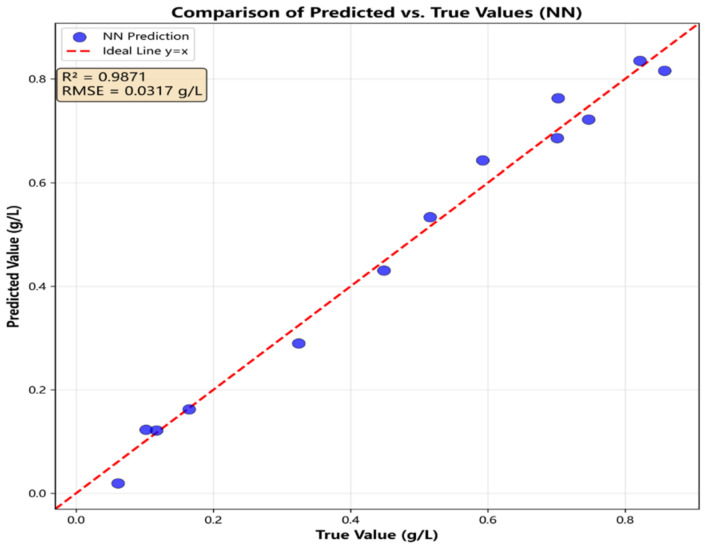
Scatter plot of predicted versus offline measured NH_4_^+^ concentrations.

The model achieved an overall R^2^ of 0.9871 and an RMSE of 0.0317 g/L. Figure 12 shows a close agreement between the predicted and offline measured values across the entire concentration range, with all points lying near the ideal y = x line. These results demonstrate that the lightweight neural network, built upon the steady-state peak features, provides accurate predictions that meet the requirements for online monitoring of NH_4_^+^ concentration.

### 3.4. Model Comparison Results

**Figure 13:**
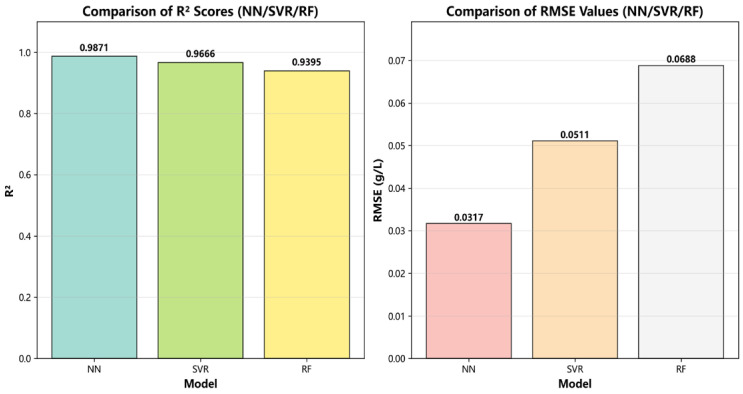
Comparison of prediction performance among different models (R^2^ and RMSE).

To evaluate the superiority of the proposed lightweight neural network model, we compare it with SVR (Support Vector Regression) and RF (Random Forest Regression) models using the same data and input features, all evaluated under the same LOOCV framework. The hyperparameters for the SVR and RF models were optimized via grid search, while the architecture and hyperparameters of the lightweight neural network were preset to prevent overfitting, given the small sample size. The results are listed in Table 1 and Figure 13.

The proposed lightweight neural network exhibits two key advantages:

- Prediction accuracy: it outperformed SVR and RF, achieving the best R^2^ and RMSE under LOOCV.
- Model robustness: its simple architecture and minimal parameters (217) inherently reduce overfitting risks, ensuring stable and reliable predictions.

In summary, the experimental results verify the effectiveness of the system solution proposed in our study from all aspects. The five-phase gas switching strategy guarantees long-time signal stability (CV < 3%). The lightweight neural network model constructed based on the characteristics of steady-state peak signals has high-precision (R^2^ = 0.9871) real-time online prediction capability (prediction delay < 1 s) for the concentration of NH_4_^+^, and this property is better than traditional machine learning models.

## 4. Discussion

This study demonstrates that the volatile organic compound (VOC) fingerprint in fermentation off-gas enables accurate, non-contact monitoring of NH_4_^+^ concentration. The high prediction accuracy (R^2^ = 0.9871) of the developed model, supported by the strong correlation (average Pearson’s r = 0.923) between the sensor response features and NH_4_^+^ concentration, jointly confirms the core mechanism proposed in the Introduction: NH_4_^+^-driven metabolic changes are specifically mapped to the off-gas VOC profile. This validates the off-gas VOC fingerprint as a direct reporter of the underlying metabolic state and provides a novel technical pathway through which to overcome the inherent lag and contamination risks of traditional offline methods.

The reliability of this approach is underpinned by converging evidence from metabolism, engineering, and data. The established correlation is grounded in a stable biological mechanism: NH_4_^+^ metabolism is closely linked to core energy pathways [1], providing a theoretical basis for a consistent VOC fingerprint under standardized processes. Our electronic nose system, engineered specifically for signal stability, reliably monitored this biological consistency. The core hardware innovation—the five-phase gas switching strategy—effectively suppressed sensor drift, maintaining a baseline coefficient of variation (CV) below 3% for all sensors throughout the experiment and enabling the extraction of robust features. Crucially, the model’s high-accuracy predictions are biologically meaningful. The independently predicted NH_4_^+^ dynamics—accurately tracing the characteristic consumption trend from approximately 0.8 g/L to 0.1 g/L—closely align with the metabolic pattern described in the literature [1] and the offline measurement range. This confirms that the system captures and quantifies a core reproducible metabolic process.

The system’s performance stems from this integrated hardware–software co-design, which systematically addresses the dual industrial demands for long-term signal stability and real-time prediction accuracy.

The boundaries of this approach and potential future research directions are discussed in the following section.

### Limitations and Future Perspectives

The high accuracy of the non-contact monitoring system is demonstrated under specific, well-controlled fermentation conditions. This is because the model’s validity is intrinsically linked to a stable process, with its training data derived from gentamicin fermentations where critical parameters (e.g., dissolved oxygen, temperature, pH) were rigorously maintained [1]. Consequently, the model’s capability is bounded, to some extent, by this stable operating regime.

Future work should prioritize the following directions: First, multi-batch fermentation experiments should be conducted to rigorously validate the repeatability and robustness of the model across different production cycles. Second, the sensing mechanism should be validated by identifying the key VOC species that underlie the observed sensor response.

## Figures and Tables

**Table 1:** Performance comparison of different prediction models for NH_4_^+^ concentration prediction in gentamicin fermentation.

Model	R^2^	RMSE (g/L)
Neural Network	0.9871	0.0317
SVR	0.9666	0.0511
RF	0.9395	0.0511

## Data Availability

The data presented in this study are openly available in Zenodo at https://doi.org/10.5281/zenodo.19853025.

## Abbreviations

The following abbreviations are used in this manuscript:

NH_4_^+^	Ammonium Nitrogen
MOS	Metal–Oxide–Semiconductor
RMSE	Root Mean Square Error
VOCs	Volatile Organic Compounds
RUP	Rational Unified Process
SVR	Support Vector Regression
RF	Random Forest Regression
LOOCV	Leave-One-Out Cross-Validation
